# Activation of D1/5 Dopamine Receptors in the Dorsal Medial Prefrontal Cortex Promotes Incubated-Like Aversive Responses

**DOI:** 10.3389/fnbeh.2017.00209

**Published:** 2017-10-31

**Authors:** Fernando Castillo Díaz, Cecilia P. Kramar, Micaela A. Hernandez, Jorge H. Medina

**Affiliations:** ^1^Instituto de Biología Celular y Neurociencias, Facultad de Medicina, University of Buenos Aires, Buenos Aires, Argentina; ^2^Departamento de Fisiología Facultad de Medicina, University of Buenos Aires, Buenos Aires, Argentina

**Keywords:** mPFC, dopamine receptors, conditioning experiments, aversive memory, dopamine agonist, dopamine antagonists

## Abstract

It is well established that neurons of the mammalian medial prefrontal cortex (mPFC) modulate different behavioral outputs, including several memory types. This behavioral modulation is, at least in part, under the control of the D1-like Dopamine (DA) receptor (D1/5R) which comprises D1 and D5-specific subtypes (D1R and D5R, respectively). Here, combining a set of behavioral assays with pharmacology, we determined whether the activation of D1/5R in the mPFC during almost neutral or weak negative-valence experiences induces aversive behaviors. The intra mPFC bilateral infusion of the D1/5R agonist SKF 38393 (6.25 μg/side) immediately after exposing rats to the white compartment of a place conditioning apparatus promotes a incubated-like aversive memory when tested 7 days thereafter, but it was not seen 24 h after conditioning. No signs of fear or changes in the anxiety state were observed after the exposure to the white compartment. This aversive response is observed only when the experience paired with the mPFC D1/5R activation has a context component involved. By using specific agonists for D1R or D5R subtypes we suggest that D5R mediate the induction of the aversive behavior. No aversive effects were observed when the D1/5R agonist was infused into the dorsal hippocampus (HP), the nucleus accumbens (NAcc) or the basolateral amygdala (BLA) of rats exposed to the white compartment. Taken together, our present findings endorse the idea that activation of mPFC D1/5R is sufficient to induce incubated-like aversive memories after exposing rats to an apparent neutral or weak negative-valence environment and that mPFC might be considered a key brain region involved in providing adaptive emotional behaviors in response to an ever-changing environment.

## Introduction

The ability to differentiate between neutral, rewarding or aversive stimulus is a key feature for our survival. When this differentiation is affected, cognitive disorders emerge that affect our quality of life (Puig et al., 2014). Diseases such as schizophrenia, depression, or anxiety disorders are characterized by abnormal emotional, cognitive or personality states. All these affections share a deregulatory key factor, which is an imbalance in the dopaminergic system (Grace, 1991; Robbins, 2000; Arnsten, 2009).

Dopamine (DA) neurotransmission is thought to play a role in associative learning and memory (Goldman-Rakic et al., 2004; Lisman and Grace, 2005; Puig et al., 2014), it participates in the processing of emotional salient stimuli and aversive signals in the environment (Lammel et al., 2014; Pignatelli and Bonci, 2015), and it is well established that aversive or stressful events excite ventral tegmental (VTA) DA neurons and cause DA release in the mPFC (Abercrombie et al., 1989; Bassareo et al., 2002; Lammel et al., 2014). Moreover, learning and memory impairments found in psychiatric and neurological disorders are generally associated with abnormalities in the mPFC dopaminergic transmission (Floresco et al., 2006). Within the mPFC, cognitive (Goldman-Rakic et al., 2004; Puig et al., 2014) and emotional processes are under the control of the DA transmission (Pezze et al., 2003; Lauzon et al., 2009; Gonzalez et al., 2014). D1-like receptors (comprising D1R and D5R subtypes) in the mPFC have been shown to modulate working memory (Goldman-Rakic, 1995), object recognition memory (Rossato et al., 2013), inhibitory avoidance and conditioned taste aversion (CTA) memories (Gonzalez et al., 2014, 2015), spatial and non-spatial memories (Clausen et al., 2011) and associative learning (Puig and Miller, 2012). However, in recent years, it has been observed varied responses after activation of these receptors, which led to propose that D1R and D5R could be regulating different phenomena (O’Sullivan et al., 2004; Hansen and Manahan-Vaughan, 2012). Cortical expression of the D5R is higher than D1R subtype, and pharmacological studies demonstrated that D5R has over a 10-fold higher affinity for DA than D1R subtype (Sunahara et al., 1991; Weinshank et al., 1991).

Due to the relevance of the mPFC dopaminergic signaling on strong negative-valence experiences, we aim to elucidate if the merely activation of the mPFC D1/5R would be able to induce an aversive behavior when it is paired with an otherwise neutral or slightly negative-valence experience. Using a combination of behavioral and pharmacology experiments, we report that the activation of the D1/5R in the mPFC produces an aversive behavior when it is paired with a slightly aversive stimulus. Moreover, this mechanism appeared to be mediated by the D5R subtype and has a incubated-like expression, since the effect is only observed when memory is tested at 7 days, but not at 24 h, after training.

## Materials and Methods

### Animals

A total of 372 Sprague-Dawley rats (Faculty of Veterinary Science, Argentina) were housed five per cage in a vivarium maintained on a reversed 12-h light-dark cycle (lights off at 0700 h) at a constant temperature of 21°C. Experimental procedures followed the guidelines, and were approved by the Animal Care and Use Committees of the University of Buenos Aires (CICUAL). The protocol was approved by the same committee. Each experiment involves an independent group of animals.

### Drugs

Cocaine hydrochloride (Coc, 20 mg/ml/kg, Laboratorios Verardo y Cia., Argentina), Lithium Chloride (LiCl, 150 mg/ml/kg, Cicarelli, Argentina) and the DA D1/5R antagonist SCH 23390 hydrochloride (SCH, 1.5 μg/μl, Sigma-Aldrich) were all dissolved in sterile 0.9% physiological saline. The DA D1/5R agonist SKF 38393 hydrochloride (SKF, 12.5 μg/μl, Sigma-Aldrich), the selective DA D1R agonist SKF 83822 hydrobromide (SKF 83822, 1 μg/μl, Sigma-Aldrich) and the selective DA D5R agonist SKF 83959 hydrobromide (SKF 83959, 10 μg/μl, Sigma-Aldrich) were all dissolved in sterile 0.9% physiological saline supplemented with DMSO (10% final concentration, Ernesto van Rossum y Cia., Buenos Aires, Argentina). The doses utilized were determined based on previous studies showing the effect of each compound on learning or behavioral performance (Majchrzak and Di Scala, 2000; Lima et al., 2009; Kramar et al., 2014).

### Surgical and Intracerebral Infusion Procedures

Each rat was anesthetized with a mix of ketamine (85 mg/kg) and xylazine (10 mg/kg) administered intraperitoneally (i.p.) and placed in a stereotaxic frame. The skull was exposed and leveled (flat skull, lambda and Bregma at the same elevation degree) 22-G guide cannulae for intracerebral infusions were bilaterally implanted aimed at different structures. The stereotaxic coordinates used were as follows for the different structures: For mPFC: AP +3.20 mm/L ±0.75 mm/DV −3.20 mm; for nucleus accumbens (NAcc): AP +1.5 mm/L ±1.2 mm/DV −1.2 mm, for hippocampus (HP): AP +3.90 mm/L ±3.00 mm/DV −3.00 mm and for basolateral amygdala (BLA): AP −2.8 mm/L ±4.6 mm/DV−6.6 mm from Bregma; (Paxinos and Watson, 2004; Supplementary Figure S1). Cannulas were fixed to the skull with acrylic cement. After surgery, animals were injected with a single dose of meloxicam (0.2 mg/kg) as analgesic and animals were left on their homecage to recover for 1 week. For intracerebral infusions, 30-G needles connected to Hamilton syringes were used. The infusions were always bilateral with 1 μl per side as volume infusions for HP and 0.5 μl for the other structures (injection rate: 1 μl/30 s). The needle was left in place for an additional minute after infusion to allow diffusion and to prevent reflux. At the end of each experiment cannulae placement was verified by infusions of 1 μl of 4% methylene blue in saline for HP and 0.5 μl for the other structures. Animals were killed after 15 min by decapitation and histological localization of the infusion site was established. The extension of the dye infused was taken as indicative of the presumable diffusion of the drugs previously given to each animal. Infusions spread with a radius of ranging from 1 mm^3^ to 1.5 mm^3^ depending on the volume infused (Gonzalez et al., 2014; Tomaiuolo et al., 2014). Animals with both cannulae in the correct place were included in the study.

### Behavioral Paradigm

**Figure 1:**
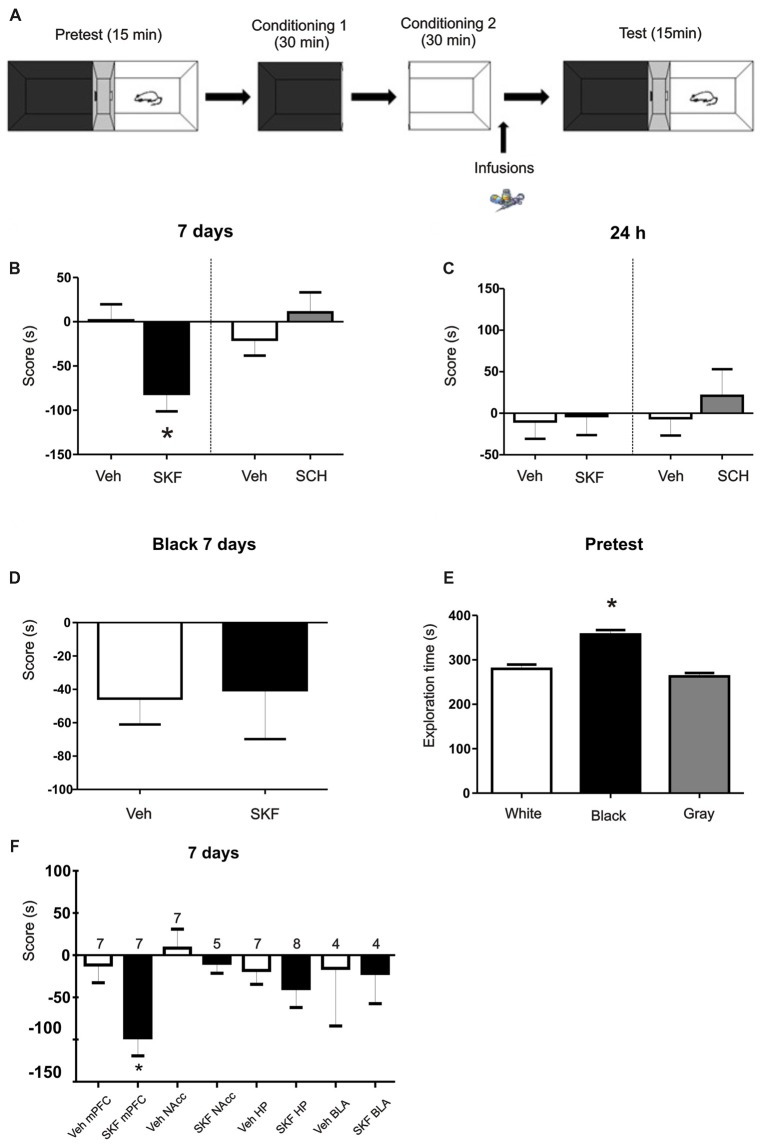
Infusion of D1/5R agonist SKF 38393 in medial prefrontal cortex (mPFC) produces an aversive behavior. **(A)** Schema of the protocol used. **(B)** Animals were infused with the D1/5R antagonist SCH 23390, with the D1/5R agonist SKF 38393 or their respective vehicles in the mPFC region immediately after the exposure of the white compartment. When animals were tested 7 days later, animals infused with SKF 38393 show an aversive behavior regarding the white compartment (Veh vs. SKF *p* = 0.0074, *n* = 9). **(C)** No effect of any of the drugs was observed when animals were tested at 24 h (*p* > 0.05, *n* = 6). **(D)** Infusions of SKF 38393 immediately after the black compartment exposure. Test was performed 7 days later. No aversive effect was found (*p* > 0.05, *n* = 12). **(E)** Scores for the pre-test phase of the protocol. Animals show a natural preference for the black compartment (*p* < 0.001, black vs. white; black vs. gray, *n* = 39). **(F)** Animals were infused with the D1/5R agonist SKF 38393 or vehicle in the hippocampus (Hp), nucleus accumbens (NAcc) or basolateral amygdala (BLA) region immediately after exposure to the white compartment of the apparatus. There is no effect of the D1/5R activation in either structure when test was performed 7 days later (*p* > 0.05, *n* is indicated on each column). *p < 0.05.

Place conditioning experiments were carried out using a three-compartment apparatus; the center compartment was a short connecting passageway between two other compartment. One of them had black walls, white square patterns and grid floor whereas the other one had white walls, black lines pattern and perforated floor. The size of the squares is 4 × 4 cm and the stripes are 1.8 × 30 cm. There are 44 squares distributed in the black compartment (14 on the longest walls and 8 on the shortest) separated 1.5 cm from each other and 20 stripes in the white compartment (6 on the longest walls and 4 on the shortest) separated approximately 4 cm from each other. The perforated floor has 270 holes with a diameter of 6 mm each separated by 1 cm from each other. The grid floor has 18 smooth circled rods separated by 1 cm from each other with a diameter of 6 mm each. The connecting passage had gray walls with no pattern and smooth floor. The dimensions of the compartments were 29 × 25 × 30 cm for the black and white compartments and the 10 × 25 × 30 cm for the gray corridor. The connecting passage had gray walls with no pattern and smooth floor. The dimensions of the compartments were 29 × 25 × 30 cm for the black and white compartments and the 10 × 25 × 30 cm for the gray corridor. All the experiments were independent and carried out with different groups of animals, but they shared part of the protocol, consisted on three phases (Figure 1A): a pretest phase in which the animals were allowed to explore the entire apparatus freely for 15 min and the preference for each compartment was determined measuring time spent in each compartment, a conditioning phase 24 h after the pretest in which they were restricted to one of the compartments after saline i.p. injections and, on the next day, confined to the other compartment after saline, cocaine (Figures 3A,B), or LiCl (Figures 3C,D) i.p injections, depending on the experiment. Immediately after removing them from this compartment, animals were infused with SKF 38393 (Figures 1, 3–5), SCH 23390 (Figures 1B,C), SKF 83959 (Figure 6) or SKF 83822 (Figure 6) into the mPFC, HP (Figure 1F), NAcc (Figure 1F) or BLA (Figure 1F) according to the experiment. Results were analyzed using the Score corresponding to time spent in the compartment that was followed by a drug infusion in the brain minus time spent in that compartment on the pretest. Time was measured with timers by a blind subject seated near the apparatus at a distance where rats cannot see it directly. The position of the CPP apparatus regarding the walls or the experimenter was counterbalanced, being some trials the white compartment closer to the experimenter and the others, the white compartment close to the wall. The environment where the experiment was performed consisted on a quiet room, with light gray walls, temperature and humidity controlled. The intracerebral injections were performed on a separate table, away from the CPP apparatus, while there were no animals being conditioning at that moment. The experiments using two drugs (Figures 3B,D) have only one conditioning drug (cocaine or LiCl) with its corresponding VEH and one modulating drug (SKF or SCH) with its corresponding veh. Therefore, conditioning drug and modulating drug are the two statistical factors with a 2 × 2 design. Finally, the test phase was made at 24 h or 7 days, in which the animals were allowed again to explore freely the entire apparatus for 15 min in a drug-free state, where time spent in each compartment was also measured. All the experiments were performed during the dark-cycle of the animals using a red mild light to illuminate the room. For detailed information of each experiment, please see “Supplementary Material” section. The training simply established an association between the drug and the contextual cues of one of the compartments. Whether the animal approaches or avoids the compartment in the test phase depends on whether the drug has rewarding or aversive effects. All experiments were analyzed separately, since they were performed separately, always with their corresponding controls (Veh suministration). During pretest phase, animals that spend less than 90 s in any of the compartments were excluded from the study. Two animals out of 372 were excluded for this reason. The description of the previous protocol was followed on all place conditioning experiments, except on the “homecage” experiment, explained next.

**Figure 2:**
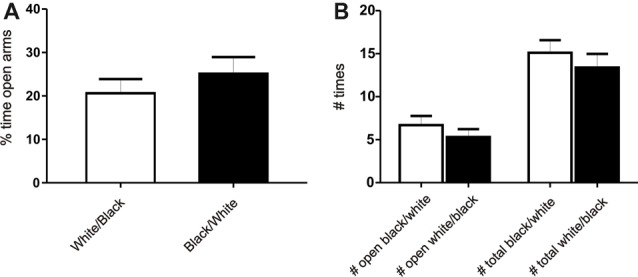
Exposure to the white compartment does not produce an anxiogenic state in rats. Animals were conditioned following the same protocol as before. Immediately after exposition to the white or black compartment, animals were tested on a Plus Maze paradigm. **(A)** Percentage of time spent in the open arms. There is no significant differences between groups (*p* > 0.05, *n* = 10). **(B)** Number of entries in the open arms and total entries into both arms. There is no significant differences in any of the variables studied (*p* > 0.05, *n* = 10).

**Figure 3:**
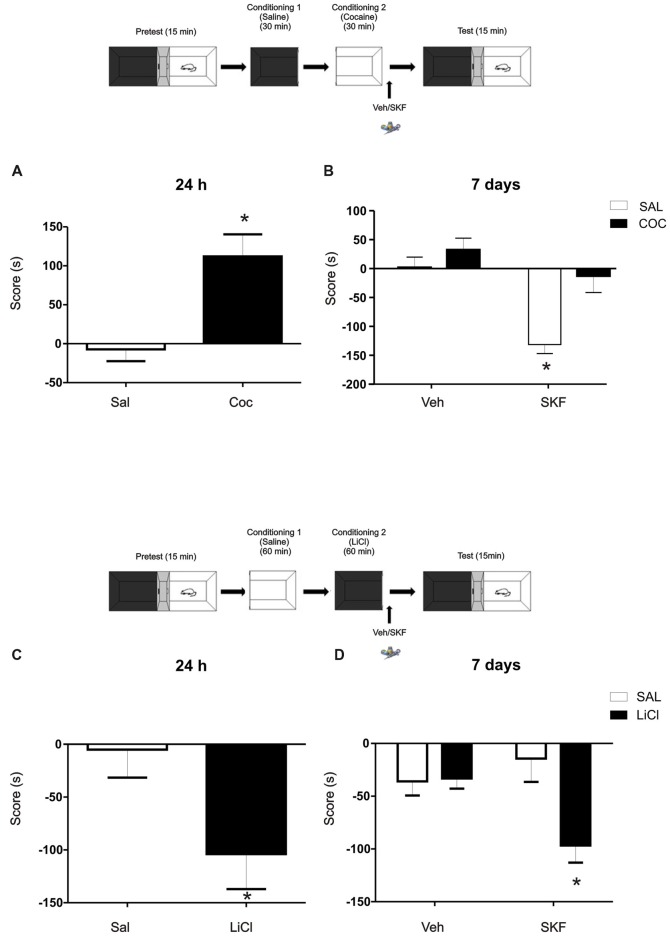
Challenging effects of Cocaine or Lithium Chloride (LiCl) reverse or promote the effects of D1/5R activation on mPFC. **(A)** A single dose of cocaine (Coc, 20 mg/kg, i.p) generates a positive conditioning to the associate, white compartment when animals are tested at 24 h (Sal vs. Coc *p* = 0.003, *n* = 18–19). **(B)** The infusion of the D1/5R agonist SKF 38393 in the mPFC after a single cocaine conditioning does not generate the aversive behavior observed at 7 days (Tukey *post hoc*, *p*_(interaction)_ = 0.0478, *n* = 10). **(C)** A single dose of LiCl (150 mg/kg i.p.) induces an aversive conditioning shown at 24 h but not at 7 days (Sal vs. LiCl *p* = 0.0032 *n* = 9). **(D)** Animals infused with the D1/5R agonist SKF 38393 or vehicle in the mPFC region immediately after the conditioning in the black compartment paired with LiCl promotes an aversive behavior 7 days later (Tukey *post hoc p*_(interaction)_ = 0.0137 *n* = 10–11). *p < 0.05.

**Figure 4:**
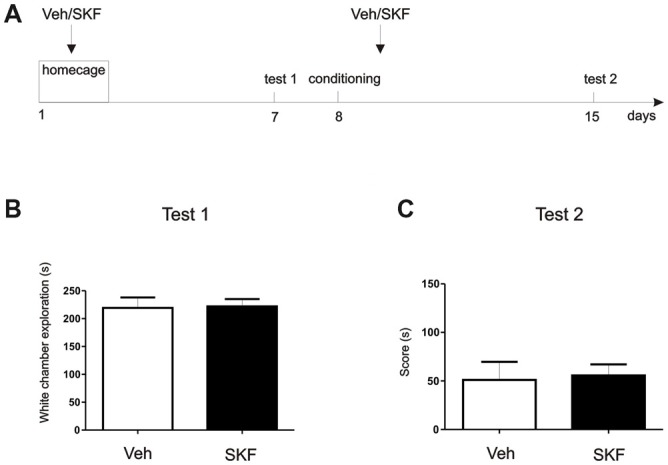
D1/D5 activation on the mPFC did not generate the aversive behavior without white compartment conditioning. **(A)** Schema of the protocol used. **(B)** Previous any exposure to the apparatus, animals were infused with D1/5R agonist SKF 38393 in the mPFC and left on their homecage. Test/Pre—test was performed 7 days later. No differences were found in the exploration time of the white compartment (*p* > 0.05, *n* = 10–11). **(C)** Animals were then conditioned only on the black compartment, and infused with D1/5R agonist SKF 38393 in the mPFC immediately after. A new test was performed 7 days later. No differences were found between groups (*p* > 0.05, *n* = 10–11).

**Figure 5:**
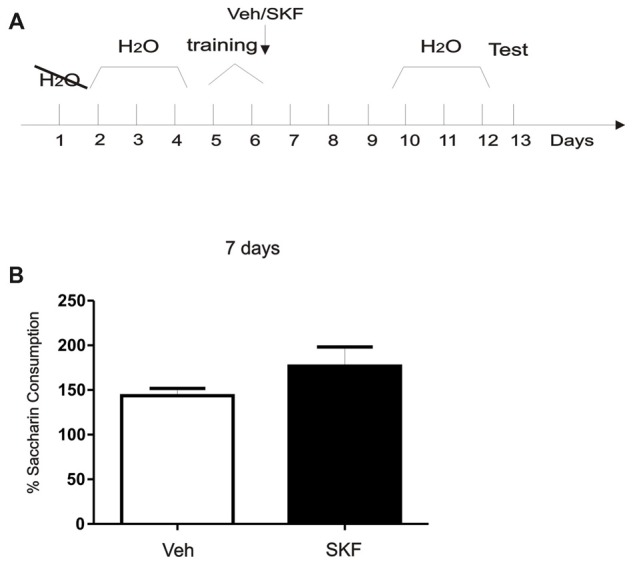
Activation of mPFC D1/5R after exposure of a neutral, context-independent paradigm did not generate an aversive response. **(A)** Schema of the modified conditioned taste aversion (CTA) protocol used. **(B)** Animals were infused with D1R agonist SKF 38393 in the mPFC immediately after being trained on saccharin consumption. Test was performed 7 days later. No significant differences were found between groups (*p* > 0.05, *n* = 6).

**Figure 6:**
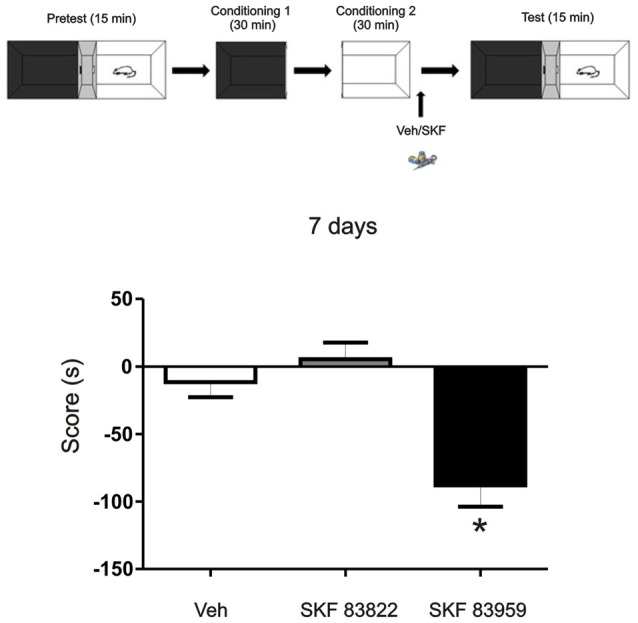
The aversive effect observed is mediated through the activation of dopamine (DA) receptor D5 in the mPFC. Animals were infused with the selective D1R dopaminergic agonist SKF 83822, with selective dopaminergic agonist SKF 83959 or Vehicle in the mPFC region immediately after the exposure to the white compartment of the apparatus. Test was performed 7 days later. Animals infused with SKF 83959 showed an aversive behavior (*p* = 0.047, *n* = 8–19) while the animals infused with SKF 83822 showed no changes in preference or aversive behavior (*p* > 0.05, *n* = 8–19). *p < 0.05.

In the “homecage” experiment, two new groups of naïve independent animals were used for this experiment. We aimed to understand if the mPFC D1/5R activation could generate aversion at 7 days without being paired to a conditioning compartment. One week after surgery animals were infused with Vehicle or SKF 38393 in the mPFC and placed back to their homecage. Seven days later animals were allowed to explore freely the CPP apparatus for 15 min (Test 1). As we did not find significant differences, we next wanted to assess if the incubated aversive effect observed in the experiment 1 (Figure 1B) also occurs even when animals were not conditioned on the white compartment. If this happens after a conditioning phase onto the black compartment, it could denote a generalization effect of the experience regarding the same neural pathway. This would mean that D5 stimulation after the black compartment conditioning leads the aversive behavior incubation that may be expressed in the white compartment, although no white compartment conditioning was made. Therefore, the following day after Test 1 animals were conditioned in the black compartment for 30 min and immediately infused with Vehicle or SKF 38393 into the mPFC. Seven days later animals were again allowed to explore freely the entire apparatus for 15 min (Test 2). Time is shown as a score of Test 2-Test 1 white compartment exploration time to determine changes in their natural behavior. Student’s *t*-test was made to compare sal and SKF groups in both tests, independently (*n* = 11).

In the positive-valence experiment, cocaine was used as a positive agent. During the conditioning phase animals were given i.p. saline injection before placement in the black compartment for 30 min on the first conditioning day and were given a Cocaine injection (20 mg/ml/kg, i.p.) before placement in the white compartment for 30 min on the following day. Immediately after removing from the white compartment, animals were infused with Vehicle or SKF 38393 into the mPFC and returned into their home cages. Control groups received saline i.p. injections both days. Animals were tested at 7 days. In the negative-valence experiment, LiCl was used as a negative agent. Animals were given an i.p. saline injection before placement in the initially non-preferred white compartment for 60 min on the first conditioning day and were given a LiCl injection (150 mg/kg, i.p.) before placement in the initially preferred black compartment for 60 min on the following day. Immediately after removing from the black compartment, animals were infused with Vehicle or SKF 38393 into the mPFC and returned into their home cages. Control groups received saline i.p. injections both days. Animals were tested at 7 days.

### Plus Maze Test

The plus-maze was made of acrylic and had two open arms (50 × 10 cm) and two enclosed arms of the same size with walls 40 cm high; it was elevated 100 cm above the ground. Each rat was placed in the central square (10 × 10 cm) and allowed 5 min to freely explore the maze. The total number of entries into the four arms, the number of entries, and the time spent in the open arms were recorded (Pellow and File, 1986). Student’s *t*-test were made for each comparison (*n* = 10).

### Taste Aversion Test

After recovery from surgery, animals were trained in a modified CTA task (CTA; Gonzalez et al., 2015; Figure 5). Briefly, animals were deprived of water for 24 h and then habituated to drink water from a graduated tube for 20 min each day for 3 days. In the training session, water was substituted with a 0.1% saccharin (Sigma-Aldrich) solution. After saccharin consumption animals were infused with Vehicle or SKF 38393 into the mPFC. After training animals received free water for 3 days and then were deprived again for 24 h followed by 3 days of limited water intake like the habituation days. Seven days after training animals were tested only once by giving them again a 0.1% saccharin solution. Saccharin consumption (in percentage) was calculated as follow: consumption in the test session × 100/consumption in the training session. Student’s *t*-test was made to compare both groups veh vs. SKF, *n* = 6, respectively.

### Statistical Analysis

Data is presented as a score in seconds (s), total exploration time, % of exploration time or number of entries for Plus Maze experiments. For the conditioning experiments the score was calculated as the time spent in the LiCl/Coc-associated compartment minus time spent in the to-be LiCl/Coc-associated compartment during the pretest. Results were presented as mean ± SEM. Data was analyzed using two way analysis of variance (ANOVA). In cases of significant interaction *post hoc* analysis were made with Tukey test when one or two factors *p-value* were significant. In cases with non-significant interaction, a main effect ANOVA was made. A result was considered significant when *p* < 0.05. Single comparisons were made with Student’s *t*-tests. All data was analyzed using Graphpad and InfoStat software.

## Results

### Activation of Dopaminergic D1/5R in mPFC Produces a Long-Lasting Aversive Behavior

We first aimed to determine if the activation of D1/5R in the dorsal mPFC has an effect on an otherwise almost neutral experience. We trained the animals on a modified place paradigm that avoids the use of rewarding or aversive agents. Animals were confined to a “white” and a “black” compartment (Figure 1A) for 30 min after a saline injection on consecutive days. A group of animals were infused in the dorsal mPFC with the D1/5R agonist SKF 38393 (6.25 μg per side) or its respective vehicle, immediately after the conditioning session in the white compartment, while another group was infused with the D1/5R agonist after the conditioning session in the black chamber. Animals were tested 7 days after the last conditioning session, where animals were placed in the three-compartment apparatus and were able to explore freely for 15 min. The group infused with the D1/5R agonist SKF 38393 after the white compartment conditioning session spent significant less time on the white compartment than control group. Moreover, they account a negative score suggesting an aversive behavior probably due to D1/5R activation (Figure 1B, Student’s *t*-test veh vs. SKF, *p* = 0.0074, *n* = 9). On the other hand, this behavior was not observed when animals were tested 24 h after the last conditioning session (Figure 1C, Student’s *t*-test veh vs. SKF, *p* > 0.05, *n* = 6), suggesting a particular effect of DA signaling on the expression of a long-lasting long term memory (LTM; tested at 7 days) but not being the case for the expression of a recent LTM (tested at 24 h). No effect was observed when animals received an infusion of the D1/5R antagonist SCH23390 (0.75 μg per side) or its respective vehicle, immediately after the conditioning session in the white compartment and were tested at 7 days (Figure 1B, Student’s *t*-test veh vs. SCH *p* > 0.05, *n* = 6 or 7, respectively) or at 24 h (Figure 1C, Student’s *t*-test veh vs. SCH *p* > 0.05, *n* = 12). Surprisingly, the group of animals infused with the D1/5R agonist immediately after the conditioning session in the black chamber did not show any aversive behavior (Figure 1D, Student’s *t*-test, veh vs. SKF *p* > 0.05, *n* = 12 or 8, respectively). This could suggest that the white compartment has different features, being somehow less attractive than the black one, even though, not sufficient to generate an aversive behavior *per se*. Indeed, an analysis of the time spent in each compartment during the pre-test phase shows that animals spent more time in the black compartment than the white one (Figure 1E, Tukey *post hoc* test after one-way ANOVA_(2,116)_; *F*_(treatment)_ = 30.37, *p* < 0.0001, black vs. white *p* < 0.05; black vs. gray *p* < 0.05, *n* = 39).

### Activation of D1/5R in Other Brain Regions Does Not Produce Any Behavioral Effect

The mPFC is part of the mesocortical circuitry and it is known to be involved in appetitive and aversive memories (Quirk and Sotres-Bayon, 2009). However, there are other structures that are intimately connected to it and that are also involved in this type of memories as the HP, NAcc and BLA. We further aimed to understand if any of these structures are also involved in the modulation of this aversive behavior. Therefore, we infused cannulated animals aiming the HP, NAcc or BLA with the D1/5R agonist SKF 38393 after the saline conditioning in the white compartment. Animals were tested at 7 days and no aversive behavior was observed, as shown in Figure 1F, suggesting that this aversive behavior could be triggered mainly by the mPFC at the doses used here (Student’s *t*-tests veh vs. SKF for each structure, *p* > 0.05, n indicated on each column).

### Exposure to a White Compartment Does Not Produce an Anxiogenic State in Rats

To determine if the exposure to any of these compartments could produce an anxiogenic state on the animals, we performed a plus-maze test (Pellow and File, 1986), in which animals were exposed to both compartment, but one group was tested after the white compartment exposure while the other was tested after the black compartment exposure. We did not find significant differences between groups, indicating no particular anxiogenic state in white compartment-exposed groups of animals (Figures 2A,B). No significant differences were found neither in the time spent nor the number of entries into the open arms between groups (Student’s *t*-test, *p* > 0.05 for each comparison, *n* = 10). In addition, in our experimental conditions no signs of freezing or changes in other fear-related measures (number of boluses or rearings) were observed during the experiments. However, it cannot be totally ruled out the possibility that a mild undetected state of anxiety is actually present in white compartment-exposed animals.

### Challenging Effects of Cocaine or LiCl Reverse or Promotes the Effects of D1/5R Activation on mPFC

To further analyze the idea of the negative-valence component that the white compartment could have, we decided to challenge this negative-valence by reversing this effect with a rewarding agent (cocaine 20 mg/kg, i.p; Kramar et al., 2014). Cocaine generates a preference for the paired compartment 24 h after conditioning (Figure 3A, Student’s *t*-test, sal vs. coc *p* = 0.003, *n* = 18 or 19 respectively). Animals were conditioned as previously, except that now a group of animals received an injection of cocaine (20 mg/ml/kg i.p.) prior being confined on the white compartment. Immediately after conditioning, animals were infused with veh or SKF 38393. No effect of the D1/5R agonist infusion on cocaine group was observed when animals were tested at 7 days and again, the aversive response was observed on the saline group infused with SKF (Figure 3B, Tukey *post hoc* after two-way ANOVA_(1,35)_; *F*_(conditioned drug)_ = 12.02, *p* = 0.0014, *F*_(modulating drug)_ = 16.96, *p* = 0.0002, *F*_(interaction)_ = 4.205, *p* = 0.0478, *n* = 10 for each group).

In accordance with this rationale, providing an aversive component to the naturally non-aversive environment as is the black compartment, infusion of the D1/5R agonist SKF 38393 following conditioning on the black compartment would cause the same incubated aversive LTM observed in animals conditioned in the white compartment. We worked with a previously developed one-trial conditioned place aversion protocol (Kramar et al., 2014) in which the animals were conditioned in the black compartment with a low dose of Lithium Chloride (LiCl), an aversive agent known to be capable of establishing taste and place avoidance (Nachman, 1963; Tenk et al., 2005), as our negative reinforcement. The one-trial version is considered a weak training that establishes a relatively weak memory for the LiCl-compartment association, where animals normally show memory retention at 24 h (Figure 3C, Student’s *t*-test sal vs. LiCl *p* = 0.032, *n* = 9) but not at 7 days (Figure 3D, vehicle groups; Kramar et al., 2014). In this case, a group of animals received a LiCl injection (150 mg/kg, i.p.) before black compartment conditioning. Animals were infused into the mPFC with the D1/5R agonist SKF 38393 immediately after conditioning on the black compartment and tested 7 days later. The activation of the mPFC D1/5R promoted a long-lasting aversive memory regarding the black compartment (Figure 3D, Tukey *post hoc* after two-way ANOVA_(1,38)_; *F*_(conditioning drug)_ = 5.860, *p* = 0.0204; *F*_(interaction)_ = 6.682, *p* = 0.0137, *n* = 10 for veh groups and *n* = 11 for SKF groups).

### The Effect Observed Is Due to an Enhancement of an Aversive Contextual Component

To better understand the behavioral outcome we were observing, we wanted to determine if this aversive behavior was exclusively a pharmacological effect or if it had a contextual component. We perform a “homecage” experiment in which at first, animals that had never been exposed to the three-compartment apparatus were infused with D1/5R agonist SKF 38393 or Veh in the dorsal mPFC (Figure 4A, Schema of the protocol used). Following infusion, animals were returned to their home-cage. Seven days post infusion, animals were placed in the apparatus and time spent in the white compartment was measured. There were no significant differences between exploration times of each group (Figure 4B, Student’s *t*-test veh vs. SKF, *p* > 0.05, *n* = 10 or 11, respectively). On the second phase of this experiment we aimed to understand if a generalization effect occurred, or if the D1/5R agonist infusion should be paired with the white compartment. Briefly, we conditioned the animals only in the black compartment and they received an infusion of D1/5R agonist SKF 38393 or Veh in the mPFC following conditioning. Animals were tested 7 days later. Again, we did not find significant differences between groups (Figure 4C, Student’s *t*-test veh vs. SKF, *p* > 0.05, *n* = 10 or 11, respectively). These results show that the effect observed is probably due to an enhancement of an aversive contextual component, rather than merely generalization of an indistinct aversive state. Therefore, the animals should have been previously exposed to the context in order to obtain a long-lasting aversive behavior.

### Activation of mPFC D1/5R after Exposition of a Neutral, Context-Independent Paradigm Did Not Generate an Aversive Response

Previous results suggest that normal maintenance of CTA memory lasting 20 days requires an early post-trainingphase of D1/5R signaling in the mPFC (Gonzalez et al., 2014). We asked what would be the effects of D1/5R activation in mPFC immediately after training in a completely neutral and context-independent paradigm using a CTA protocol without conditioning with an aversive agent as LiCl (Figure 5A, Schema of the protocol used). When we infused the D1/5R agonist in the mPFC immediately after training and tested 7 days later, no significant differences between groups were found (Figure 5B, Student’s *t*-test, veh vs. SKF, *p* > 0.05, *n* = 6). These results indicate that activation of mPFC D1R after exposure of a neutral, context-independent paradigm like the one we performed here did not generate an aversive response.

### The Incubated Aversive Effect Observed Is Mediated through the Activation of Dopamine Receptor D5 in the mPFC

DA D1/5Rs comprising of D1R and D5R subtypes are intimately implicated in dopaminergic regulation of fundamental neurophysiologic processes such as mood, motivation, cognitive function and motor activity. To understand more about the mechanisms underlying the aversive behavior observed here, we aimed to determine the involvement of the D1R or D5R subtype activation, on this long term aversive behavior. We trained the animals using the same protocol as above (see Figure 1A) but in this case, one group of animals was infused with the specific D1-subtype receptor agonist SKF 83822 and the other one received the specific D5-subtype receptor agonist SKF 83959. Animals were tested 7 days later. We found that the aversive behavior observed 7 days after training is mediated by D5R signaling while D1R activation had no effect (Figure 6, Tukey *post hoc* analysis after One-way ANOVA_(2,37)_, *F*_(treatment)_ = 10.88, *p* = 0.0002, SKF83959 vs. veh *p* < 0.05, SKF 83959 vs. SKF 83822 *p* < 0.05, *n* = 19 for veh, *n* = 11 for SKF 83822, *n* = 8 for SKF 83959).

## Discussion

Most experiences have an aversive or rewarding component, even if it is so slight that does not affect the behavioral outcome. In this study we aimed to study what would be the implications of D1/5R activation of the mPFC when the experience has no significant valence, it means, no apparent aversive or rewarding component strong enough to produce a change in behavior.

The main finding of the present study is that activation of D1/5R in the mPFC promotes incubated-like aversive responses. This is consistent with the idea that “the mPFC is conceived as a network mapping events within a given spatial and emotional context with the most adaptive action and/or emotional responses” (Euston et al., 2012). The D1/5R agonist SKF 38393 infused into the mPFC immediately after rats were exposed to a mild, brighter environment (because of the light reflection on the white walls) with a perforated floor type (we cannot rule out the possibility that different tactile experience’s on both chambers could also be an aversive factor) promoted the acquisition of a persistent contextual aversive memory that is noticeable 7 days after training. Interestingly, no evidence of place aversion was found 24 h after the administration of the drug. When drugs are infused in the brain they might affect different memory phases depending on the moment in which they were infused. In the present work the infusions were made immediately after conditioning and therefore they may affect the formation and/or persistence of memory storage. Dopaminergic system is involved in a mechanism known as memory persistence (Medina et al., 2008; Rossato et al., 2009). It refers to as the process by which memories can last longer on time. It has been proved that during an inhibitory avoidance training (IA) the VTA becomes active and therefore releasing DA in certain structures as the mPFC and the HP that are involved in fear memory processing (Rossato et al., 2009; Gonzalez et al., 2014). VTA-mPFC DA signaling at the moment of the training is decisive for the storage of these persistent memories (Gonzalez et al., 2014). Therefore it is not surprising that an imbalance in this system could impair the normal functioning of this memory persistence machinery. In fact, blocking them PFC dopaminergic system immediately after strong IA training impairs memory retention when it is measured at 7, but not at 2 days. It seems that this aversive process is not generated immediately after the agonist infusion. On the contrary, a few days are required to evidence it. Activation of mPFC DA activity after a weak IA training promotes the persistence of this memory, also suggesting the importance of mPFC DA signaling on memory storage (Gonzalez et al., 2014). In these experiments the aversive component was strong enough to generate a memory that lasts at least 24 h. What is interesting is that the affected outcome is observed in the animal behavior at longer times, 7 days or longer, after the manipulation of them PFC DA system. This may be because we are affecting the persistence phase of this memory, without manipulating its formation. In our present study the activation of D1/5R in the mPFC associated with a mild aversive environment provokes a incubated-like behavioral aversion that also needs time to be built up.

On the other hand, the infusion of SKF38393 in the mPFC produced no signs of aversion when rats were exposed to a dark environment, which appears to havemore neutral-valence components than the white one. However, when we paired the black compartment with an aversive component (a single dose of LiCl), coupled to D1/5R activation, a delayed aversive response was obtained. This would seem to be not only the result of the value of the experience, but also the type of learning. When we worked with a context-independent protocol, we did not observe any effect.

It has been suggested that the mPFC is an important part of the brain circuit involved in fear behavior. Within this circuit, the prelimbic (dorsal) and infralimbic (ventral) subdivisions of the mPFC are thought to exert top-down control over BLA, NAcc and periaqueductal gray matter to regulate appropriate behavioral responses including the expression and suppression of learned fear (Corcoran and Quirk, 2007; Sierra-Mercado et al., 2011; Cheriyan et al., 2016; Dejean et al., 2016). However, it has been also suggested that both subdivisions of the mPFC may act in concert (Giustino and Maren, 2015) and may influence similar populations of neurons in the BLA (Arruda-Carvalho and Clem, 2015). Previous findings suggest that fear memory conditioning is under the modulatory control of DA in the NAcc and BLA in mice (Fadok et al., 2010) and in the HP in rats (Rossato et al., 2009). However, our results showed that the activation of D1/5R in these three brain structures did not induce a contextual aversive memory (Figure 1F) like that observed in the present study, suggesting that mPFC is a key node on aversive LTM processing. Other brain regions such as the lateral habenula (LH) and the periaqueductal gray matter which receive inputs from the mPFC (Euston et al., 2012) are candidates to participate in the neural circuits involved in long-lasting aversive responses (Tomaiuolo et al., 2014).

Lesion and pharmacological inactivation of mPFC on fear behavior gave inconsistent findings (Courtin et al., 2013). The lack of consistency of mPFC lesion and inactivation studies has been attributed to differences in species, in the precise prefrontal territory targeted, in the type of training or in the time when the lesion or the inactivation are performed in relation to the training. However, it has been recently demonstrated that dorsal mPFC participates in the encoding, consolidation and reconsolidation of aversive memories (Sharpe and Killcross, 2014; Gonzalez et al., 2014). In this context, optogenetic interrogation of dorsal mPFC neuronal assemblies initiated freezing behavior in unconditioned animals, modulated fear expression in previously conditioned animals (Courtin et al., 2013), promoted the acquisition of conditioned fear (Yau and McNally, 2015) and controlled the precise timing of fear responses (Dejean et al., 2016). Therefore, the role of the mPFC is not limited to regulate fear expression but also plays an important role in the encoding of fear behavior itself (Arruda-Carvalho and Clem, 2014).

DA neurotransmission in the mPFC plays a role in the processing of emotional information, fear encoding and expression (Pezze et al., 2003; Lauzon et al., 2009; Arruda-Carvalho and Clem, 2014; Gonzalez et al., 2014) contextual information (Seamans et al., 1998), and aversion inputs coming from the LH (Lammel et al., 2012; Stamatakis and Stuber, 2012) through activation of D1/5Rs. It is well-established that aversive and stressful events can excite VTA DA neurons and can cause DA release in target structures (Frankland and Bontempi, 2005; Lammel et al., 2014). Strong aversive stimuli cause modifications on synapses on the medial VTA DA neurons projecting to mPFC (Lammel et al., 2011) and receiving from LH (Pignatelli and Bonci, 2015). Optical stimulation of the LH terminals in the VTA produces conditioned place aversion, and it is blocked by the D1R antagonist SCH 23390 administration (Lammel et al., 2012). In humans, an aversive experience activates VTA and LH and increases the functional connectivity between LH, VTA and mPFC (Hennigan et al., 2015). In this context, would be interesting to further study LH-VTA-mPFC circuitry regarding incubated-like aversive responses as the ones found by activating D1/5R in the mPFC.

Given that SKF 38393 does not discriminate between D1R and D5R subtypes, we used SKF 83822 who selectively recognizes D1R-linked to adenylyl cyclase (Undieh, 2010) and SKF 83959 which is an atypical DA receptor agonist that selectively activates PLC mediated by the D5R, but does not activates adenylyl cyclase-linked D1R (Sahu et al., 2009; Undieh, 2010). The D5R is more abundant than the D1R in the mPFC (Luedtke et al., 1999). Our findings support the idea that the activation of D5R is involved in the promotion of a incubated-like spatial aversive memory. It has been shown that strong footshock training in rats using a fear conditioning task induces an enhancement of fear between recent and remote memory retention (Poulos et al., 2016). All together this suggests that a D5R activation signaling could be involved in a mechanism of fear incubation during time.

In mPFC the activation of D5R by the atypical DA agonist SKF 83959 enhances BDNF-mediated signaling in rats and in mice gene-deleted for the D1R but not for the D5R (Perreault et al., 2012). Given the known facilitatory effect of BDNF on memory persistence in cortical regions (Bekinschtein et al., 2007; Martínez-Moreno et al., 2011; Katche and Medina, 2015), which is under the control of dopaminergic neurotransmission (Rossato et al., 2009), we are tempting to suggest that the promotion of an aversive memory 7 days after the activation of D1R in the mPFC might be due to the involvement of BDNF signaling. Further experiments will be needed to test this assumption.

In conclusion, our present findings indicate that the activation of D1/5R in the dorsal mPFC induces incubated aversive outcome in response to mild negative-valence experiences. It also suggests that an unbalanced D5R subtype activation in dorsal mPFC might provoke aberrant lasting avoidance behaviors. This could have important implications on the study of emotional processes, aversive learning and even so, psychiatric diseases where the DA system is clearly affected.

## Author Contributions

JHM, CPK and FCD planned the design of the experiments and wrote the article. FCD, CPK and MAH carried out the experiments.

## Conflict of Interest Statement

The authors declare that the research was conducted in the absence of any commercial or financial relationships that could be construed as a potential conflict of interest.
