# Supplementary material for: Activation of D1/5 Dopamine Receptors in the Dorsal Medial Prefrontal Cortex Promotes Incubated-Like Aversive Responses
Source: Front Behav Neurosci. 2017 Oct 31;11:209. doi: 10.3389/fnbeh.2017.00209 (PMC5674926; doi:10.3389/fnbeh.2017.00209)
Supplement: Supplementary file 1 [file Data_Sheet_1.docx]

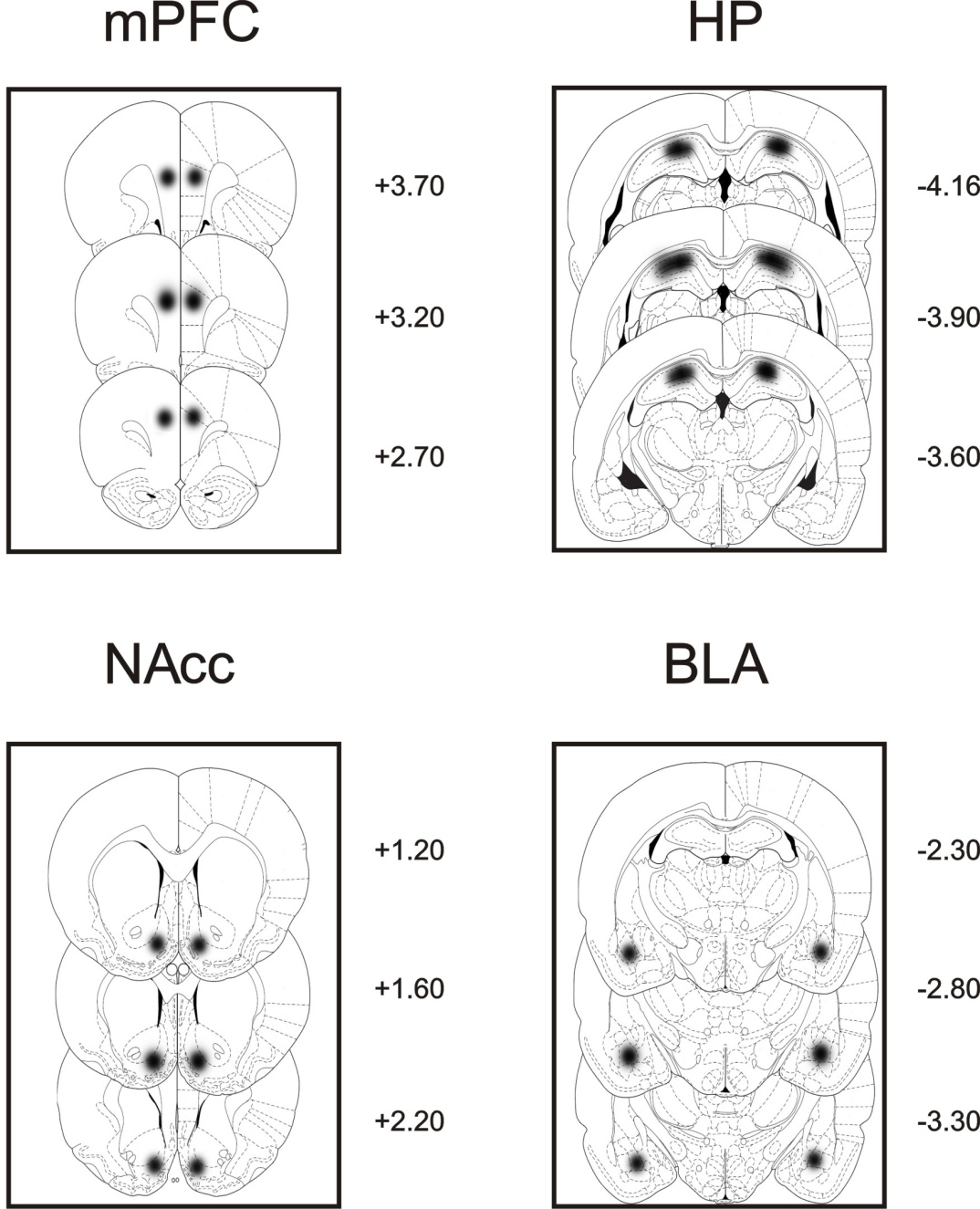


Supplementary Figure 1. **Location and** **extension of the infusions performed.** The extension was verified at the end of each experiment by infusing 0.5 µl (1µl for HP region) methylene blue per side. The drawings were adapted from Paxinos and Watson, 2004.

Detailed experimental design

Experiment 1 (Fig. 1B)

Animals were allowed to explore freely the entire CPP apparatus for 15 min. The next day animals were injected with saline (i.p.) and placed in the black compartment for 30 min. The following day animals were injected with saline (i.p.) and placed in the white compartment for 30 min. Immediately after this a group of animals were infused with Veh and other group infused with SKF in the mPFC (n = 9 for each group). Animals were tested by exploring the entire CPP apparatus at 7 days. Data was analyzed with Student’s t-test to compare both group scores.

Experiment 2 (Fig. 1B)

Another group of animals were allowed to explore freely the entire CPP apparatus for 15 min. The next day animals were injected with saline (i.p.) and placed in the black compartment for 30 min. The following day animals were injected with saline (i.p.) and placed in the white compartment for 30 min. Immediately after this time a group of animals were infused with Veh and other group infused with SCH in the mPFC (n = 6 or 7 respectively). Animals were tested by exploring the entire CPP apparatus at 7 days. Data was analyzed with Student’s t-test to compare both group scores.

Experiment 3 (Fig. 1C)

Another group of animals were used for the same as experiment 1 but both groups were tested at 24 h. Data was analyzed with Student’s t-test to compare both group scores (n=6 for veh and SKF).

Experiment 4 (Fig. 1C)

Another group of animals were used for the same as experiment 2 but both groups were tested at 24 h. Data was analyzed with Student’s t-test to compare both group scores (n=12 for veh and SCH).

Experiment 5 (Fig. 1D)

Another group of animals was used for the same as experiment 1 but now, the first conditioning session was made in the white compartment and the second one was made in the black one. Immediately after this a group of animals were infused with Veh and other group infused with SKF in the mPFC. Data was analyzed with Student’s t-test to compare both group scores (n = 12 or 8 for veh and SKF, respectively).

Fig. 1E

A set of animals used in the previous experiments was pooled to analyze their exploration times during the pretest phase for the three compartments of the apparatus. One-way ANOVA was made to compare these times. Tuckey post-hoc was made after ANOVA. (n = 39).

Experiments 6, 7, 8 and 9 (Fig. 1F).

Another set of animals was used to do an experiment as number 1 but, immediately after the second conditioning animals were infused with Veh or with SKF in the mPFC (n = 7 for each group), HP (n = 7 for each group), NAcc (n = 7 or 5 respectively) or BLA (n = 4 for each group). Animals were tested by exploring the entire CPP apparatus at 7 days. Data was analyzed with Student’s t-test to compare each pair of scores for each structure veh vs SKF.

Experiment 10: Plus maze test (Fig. 2)

Please see materials and methods section for this experiment.

Experiment 11 (Fig. 3A)

Another group of animals were allowed to explore freely the entire CPP apparatus for 15 min. The next day animals were injected with saline (i.p.) and placed in the black compartment for 30 min. The following day animals were injected with cocaine (i.p.) and placed in the white compartment for 30 min. The following day animals were allowed to explore freely the entire apparatus for 15 min. Data was analyzed as the score for each group (Student’s t-test sal vs coc n = 18 or 19 respectively, Fig 3A).

Experiment 12 (Fig. 3B)

Another group of animals were used for a different experiment. Animals were allowed to explore freely the entire CPP apparatus for 15 min. The next day animals were injected with saline (i.p.) and placed in the black compartment for 30 min. The following day animals were injected with cocaine (i.p.) and placed in the white compartment for 30 min. Immediately after conditioning, animals were infused with veh or SKF 38393. Control groups received saline i.p. injections both days. Animals were tested at 7 days. One-way ANOVA was made to compare these three groups (n=9 for sal/veh, n=10 for coc/veh and coc/skf; Fig 3B).

Experiment 13 (Fig. 3C)

Another group of animals were allowed to explore freely the entire CPP apparatus for 15 min. The next day animals were injected with saline (i.p.) and placed in the white compartment for 60 min. The following day animals were injected with LiCl (i.p.) and placed in the black compartment for 60 min. Test was made at 24 h. Student’s t-test was made to compare both groups (Fig 3C).

Experiment 14 (Fig. 3D)

Another group of animals were allowed to explore freely the entire CPP apparatus for 15 min. The next day animals were injected with saline (i.p.) and placed in the white compartment for 60 min. The following day animals were injected with LiCl (i.p.) and placed in the black compartment for 60 min. Immediately after removing from the black compartment, animals were infused with Vehicle or SKF 38393 into the mPFC and returned into their home cages. Control groups received saline i.p. injections both days. Animals were tested at 7 days. Two-way ANOVA was made (factor 1: conditioning drug sal or LiCl, factor 2: modulating drug veh or SKF). Tuckey post hoc was made (n = 10 for veh groups and n = 11 for SKF groups).

Experiment 15 (Fig. 4) Home cage experiment.

In the “homecage” experiment, two new groups of naïve independent animals were used for this experiment. We aimed to understand if the mPFC D1/5R activation, could generate aversion at 7 days without being paired to a conditioning compartment. One week after surgery animals were infused with Vehicle or SKF 38393 in the mPFC and placed back to their homecage. 7 days later animals were allowed to explore freely the CPP apparatus for 15 min (Test 1). As we did not find significant differences, we next wanted to assess if the delayed aversive effect observed in the experiment 1 (Fig 1B) also occurs even when animals were not conditioned on the white compartment. If this happens after a conditioning phase onto the black compartment, it could denote a generalization effect of the experience regarding the same neural pathway. Therefore, the following day after Test 1 animals were conditioned in the black compartment for 30 min and immediately infused with Vehicle or SKF 38393 into the mPFC. 7 days later animals were again allowed to explore freely the entire apparatus for 15 min (Test 2). Time is shown as a score of Test 2-Test 1 white compartment exploration time to determine changes in their natural behavior. Student’s t-test was made to compare sal and SKF groups in both tests, independently (n=10 or 11, respectively).

Experiment 16 (Fig. 5)

Please see materials and methods section for this experiment.

Experiment 17 (Fig. 6)

Animals were allowed to explore freely the entire CPP apparatus for 15 min. The next day animals were injected with saline (i.p.) and placed in the black compartment for 30 min. The following day animals were injected with saline (i.p.) and placed in the white compartment for 30 min. Immediately after this time a group of animals were infused with Veh, another group infused with SKF 83822 and other with SKF 83959 in the mPFC (n = 19, 11 and 8 respectively for each group). Tuckey *post-hoc* was made after one-way ANOVA to compare the three groups.
